# Supplementary figures and images for: Sperm and northern bottlenose whale interactions with deep-water trawlers in the western North Atlantic
Source: PLoS One. 2023 Aug 23;18(8):e0289626. doi: 10.1371/journal.pone.0289626 (PMC10446179; doi:10.1371/journal.pone.0289626)

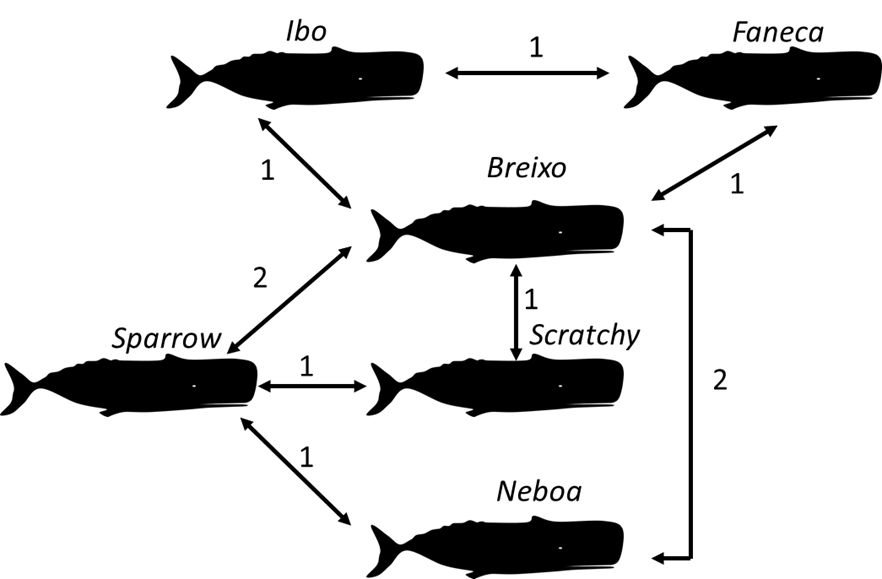

Supplement: S1 Fig — The arrows show the presence of both individuals together near the vessel, and the number of times the whales were seen together. Breixo (middle) was seen with all the other animals. (TIF) [file pone.0289626.s001.tif]

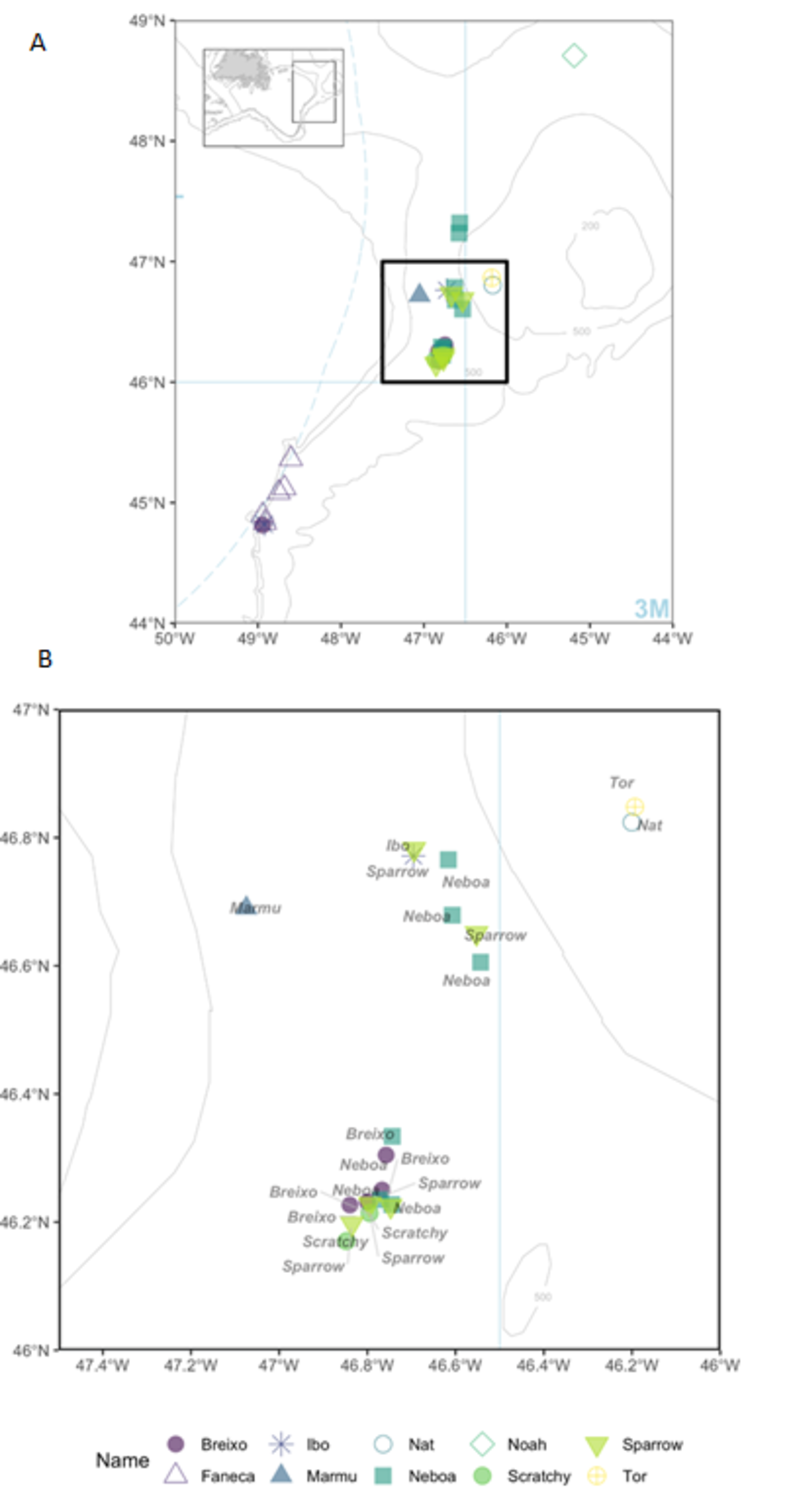

Supplement: S2 Fig — Location of sightings of individual sperm whales in (A) the entire study area and (B) an inset of concentrated sightings in the Flemish Pass. Maps were created using R [16]. The bathymetric data was obtained from the Open Government Licence–Canada [17]. (TIF) [file pone.0289626.s002.tif]
