# Supplementary material for: Sperm and northern bottlenose whale interactions with deep-water trawlers in the western North Atlantic
Source: PLoS One. 2023 Aug 23;18(8):e0289626. doi: 10.1371/journal.pone.0289626 (PMC10446179; doi:10.1371/journal.pone.0289626)
Supplement: S1 Table — Trawler activity codes: (H) Hauling (P/S) Preparing/Shooting (T) Towing. (DOCX) [file pone.0289626.s003.docx]

## Table S1. Summary of sperm whale photo id data, showing date, haul number, time, fishing area, vessel activity and speed of each encounter. Vessel activity codes: (H) Hauling (P/S) Preparing/Shooting (T) Towing

| **Name** | **ID features** | **Date** | **Haul Number** | **Time** | **Fishing Area** | **Vessel Activity** | **Accompanied**  **by** | **Distance between encounters (km)** | **Elapsed Days** | **Elapsed Hours** | **Min speed (km/h)** |
| --- | --- | --- | --- | --- | --- | --- | --- | --- | --- | --- | --- |
| *Sparrow* | scar behind dorsal fin, two little scars close to the blow hole and fluke | 7/8/2007 | 44 | 17:00 | *Playa Nueva* | H | *Breixo, Scratchy* | **-** |  |  |  |
|  |  | 10/8/2007 | 48 | 10:45 | *Playa Nueva* | H | *Neboa, Breixo* | **5** | 2.74 | 65.75 | 0.08 |
|  |  | 14/08/2007 | 55 | 9:00 | *Playa Nueva* | H | *Scratchy* | **9** | 3.93 | 94.25 |  |
|  |  | 15/08/2007 | 58 | 19:30 | *Playa Nueva* | H |  | **58** | 1.44 | 34.50 | 1.68 |
|  |  | 11/9/2007 | 125 | 18:00 | *Playa Nueva* | P/S |  | **16** | 26.94 | 646.50 | 0.02 |
| *Scratchy* | scratch in left lateral | 7/8/2007 | 44 | 17:00 | *Playa Nueva* | H | *Sparrow, Breixo* | **-** |  | 0.00 |  |
|  |  | 14/08/2007 | 55 | 9:00 | *Playa Nueva* | H | *Sparrow* | **4** | 6.67 | 160.00 | 0.03 |
| *Breixo* | right lateral scar, snout scar and fluke | 7/8/2007 | 44 | 17:00 | *Playa Nueva* | H | *Sparrow, Scratchy* | **-** |  | 0.00 |  |
|  |  | 10/8/2007 | 48 | 10:45 | *Playa Nueva* | H | *Neboa, Sparrow* | **5** | 2.74 | 65.75 | 0.08 |
|  |  | 14/08/2007 | 56 | 9:45 | *Playa Nueva* | P/S |  | **7** | 3.96 | 95.00 | 0.07 |
|  |  | 14/08/2007 | 56 | 11:00 | *Playa Nueva* | T | *Neboa* | **13** | 0.05 | 1.25 | 10.40 |
|  |  | 27/08/2007 | 86 | 10:15 | *Carson Canyon* | P/S | *Faneca, Ibo* | **235** | 12.97 | 311.25 | 0.76 |
| *Neboa* | scars in both laterals and fluke | 8/8/2007 | 45 | 18:15 | *Playa Nueva* | H |  | **-** |  | 0.00 |  |
|  |  | 8/8/2007 | 46 | 20:15 | *Playa Nueva* | T |  | **8** | 0.08 | 2.00 | 4.00 |
|  |  | 10/8/2007 | 48 | 10:45 | *Playa Nueva* | H | *Breixo, Sparrow* | **113** | 1.60 | 38.50 | 2.94 |
|  |  | 10/8/2007 | 49 | 22:00 | *Playa Nueva* | H |  | **1** | 0.47 | 11.25 | 0.09 |
|  |  | 13/08/2007 | 54 | 18:15 | *Playa Nueva* | T |  | **57** | 2.84 | 68.25 | 0.84 |
|  |  | 14/08/2007 | 56 | 11:00 | *Playa Nueva* | T | *Breixo* | **50** | 0.70 | 16.75 | 2.99 |
|  |  | 14/08/2007 | 56 | 18:15 | *Playa Nueva* | T |  | **36** | 0.30 | 7.25 | 4.97 |
|  |  | 14/08/2007 | 56 | 21:00 | *Playa Nueva* | H |  | **12** | 0.11 | 2.75 | 4.36 |
| *Faneca* | left lateral scar, fluke | 27/08/2007 | 86 | 10:15 | *Carson Canyon* | P/S | *Breixo, Ibo* | **-** |  | 0.00 |  |
|  |  | 28/08/2007 | 88 | 21:05 | *Carson Canyon* | H |  | **59** | 1.45 | 34.83 | 1.69 |
|  |  | 29/08/2007 | 90 | 9:30 | *Carson Canyon* | T |  | **54** | 0.52 | 12.42 | 4.35 |
|  |  | 29/08/2007 | 90 | 14:45 | *Carson Canyon* | T |  | **31** | 0.22 | 5.25 | 5.90 |
|  |  | 29/08/2007 | 91 | 21:15 | *Carson Canyon* | T |  | **5** | 0.27 | 6.50 | 0.77 |
| *Ibo* | scar in dorsal fin and three little depressions in the surface of the head | 27/08/2007 | 86 | 10:15 | *Carson Canyon* | P/S | *Faneca, Breixo* | **-** |  | 0.00 |  |
|  |  | 9/9/2007 | 121 | 16:30 | *Playa Nueva* | H |  | **276** | 13.26 | 318.25 | 0.87 |
| *Marmu* | fluke | 8/8/2007 | 45 | 13:23 | *Playa Nueva* | T |  | **-** |  |  |  |
| *Tor* | scar in blow hole | 29/07/2007 | 25 | 11:10 | *Playa Nueva* | H |  | **-** |  |  |  |
| *Nat* | fluke | 28/07/2007 | 23 | 10:44 | *Playa Nueva* | H |  | **-** |  |  |  |
| *Noah* | scar in left lateral and fluke | 29/08/2007 | 91 | 21:15 | *Carson Canyon* | T | *Faneca* | **-** |  |  |  |
